# Clinical significance of histopathological features of paired recurrent gliomas: a cohort study from a single cancer center

**DOI:** 10.1186/s12885-022-10484-9

**Published:** 2023-01-03

**Authors:** Cong Li, Shaoyan Xi, Yingshen Chen, Chengcheng Guo, Ji Zhang, Qunying Yang, Jian Wang, Ke Sai, Jing Zeng, Jing Wang, Zhiqiang Zhang, Chao Ke, Zhongping Chen

**Affiliations:** 1 State Key Laboratory of Oncology in South China, Collaborative Innovation Center for Cancer Medicine, Sun Yat-Sen University Cancer Center, Guangzhou, 510060 China; 2 The Second Affiliated Hospital of Guangzhou University of Chinese Medicine, Guangdong Province Hospital of Chinese Medicine, Guangzhou, 510120 China

**Keywords:** Recurrent glioma, Reoperation, Histopathology, Prognosis

## Abstract

**Objective:**

To explore the histopathological characteristics of paired recurrent gliomas and their clinical significance.

**Methods:**

Glioma patients who received both primary surgery and reoperation when recurrence at Sun Yat-sen University Cancer Center from June 2001 to June 2019 were enrolled. Clinical and pathological characteristics were analyzed retrospectively, and histopathology of reoperation specimens was divided into three categories according to tumor cell activity and the degree of necrosis: active group, low-activity group, and necrosis group.

**Results:**

A total of 89 patients were included in this study. The 2016 WHO grade of the first operation pathology and IDH1 status were related to survival time after the first operation, but there was no significant association with survival time after reoperation. The time interval between primary and reoperation was shorter for primary high-grade glioma and/or IDH1 wild-type tumor patients than for low-grade glioma and/or IDH1 mutant tumor patients (*P* < 0.001). Histopathological types of recurrent gliomas were analyzed, and 67 cases (75.3%) were classified into the active group, 14 (15.8%) into the low-activity group, and 8 (8.9%) into the necrosis group. The low-activity or necrosis group was associated with a higher radiotherapy dose and shorter operation interval. Further univariate and multivariate Cox survival analyses showed the histopathological patterns of recurrent gliomas to be related to survival time after reoperation.

**Conclusion:**

Primary WHO low grade or IDH1 mutant gliomas appeared survival benefit mainly on later recurrence, but was not a prognostic predictor following recurrence. Histopathological feature of recurrent glioma is related to previous treatment, including radiotherapy dosage and chemotherapy treatment, and is also an important independent prognostic factor for patients after reoperation.

## Introduction

Glioma is one of the most common primary malignant brain tumors, accounting for approximately 80% of primary malignant tumors in the central nervous system [1]. The median survival time of glioblastoma (GBM) patients, which is the most malignant brain tumor, is only approximately 12–15 months, even if standardized surgery, radiotherapy and chemotherapy are given [2]. Most glioma patients experience recurrence, but the diagnosis and subsequent treatment opinions of recurrent glioma are still inconsistent. Repeat surgery is an important choice for those with suspected recurrence involving increased intracranial pressure, good functional status, and amenability to surgical treatment [3]. What’s more, we may use multimodal magnetic resonance (MR) or positron emission computed tomography (PET) to differentiate whether the tumor is a true recurrence in clinical work. To date, there has been progress in molecular-level study of recurrent glioma with regard to which molecular characteristics change upon relapse, with obvious time heterogeneity for glioma [4, 5]; the histopathology of recurrent gliomas may also change. The most common phenomenon is that low-grade gliomas progress to high-grade gliomas when they recur, and some recurrent gliomas show changes induced by treatment. Therefore, for treatment of patients with recurrence who receive reoperation, we should not only focus on the pathology of the first operation and the subsequent treatment process but also pay attention to the histopathological characteristics of the second operation. Nevertheless, studies comparing differences using paired samples of primary and recurrent gliomas are relatively limited, especially for the diagnosis and clinical significance of histopathology of recurrent glioma. Given that glioma recurrence is almost inevitable and the pathological results of recurrent glioma are an important issue for further treatment, pathological assessment would play an essential role in the management of recurrent glioma, as it does for primary tumors. Therefore, in this study, we retrospectively investigated the histopathological features of recurrent glioma and evaluated clinical significance.

## Materials and methods

### Patients

A retrospective cohort study was conducted for glioma patients who received both primary surgery and a second operation when tumor local recurrence was suspected based on clinical symptoms and imaging examinations such as MRI and/or PET between June 2001 and June 2019 at Sun Yat-sen University Cancer Center. Among 1051 surgical treatment brain glioma patients, 107 glioma patients who underwent two or more craniocerebral tumor resection surgeries were collected. Excluding those whose first operation pathology was not clear (*N* = 8), those whose operation interval between two surgeries was less than 3 months (*N* = 5), and those whose first pathological diagnosis was pilocytic astrocytoma (*N* = 2), 89 patients were finally included in this study. Clinical data, including age, sex, and adjuvant treatments, were collated from the Hospital Information System (HIS) of our cancer center. The extent of tumor resection at the first surgery was recorded as gross total resection (GTR) and partial resection (PR), which included “near total” or “subtotal” resection in surgical records. Mortality and follow-up data were collected from the follow-up department of our cancer center. This study was approved by the Institutional Review Board of Sun Yat-sen University Cancer Center (approval No. B2020-314–01).

### Histopathological analysis

**Fig. 1:**
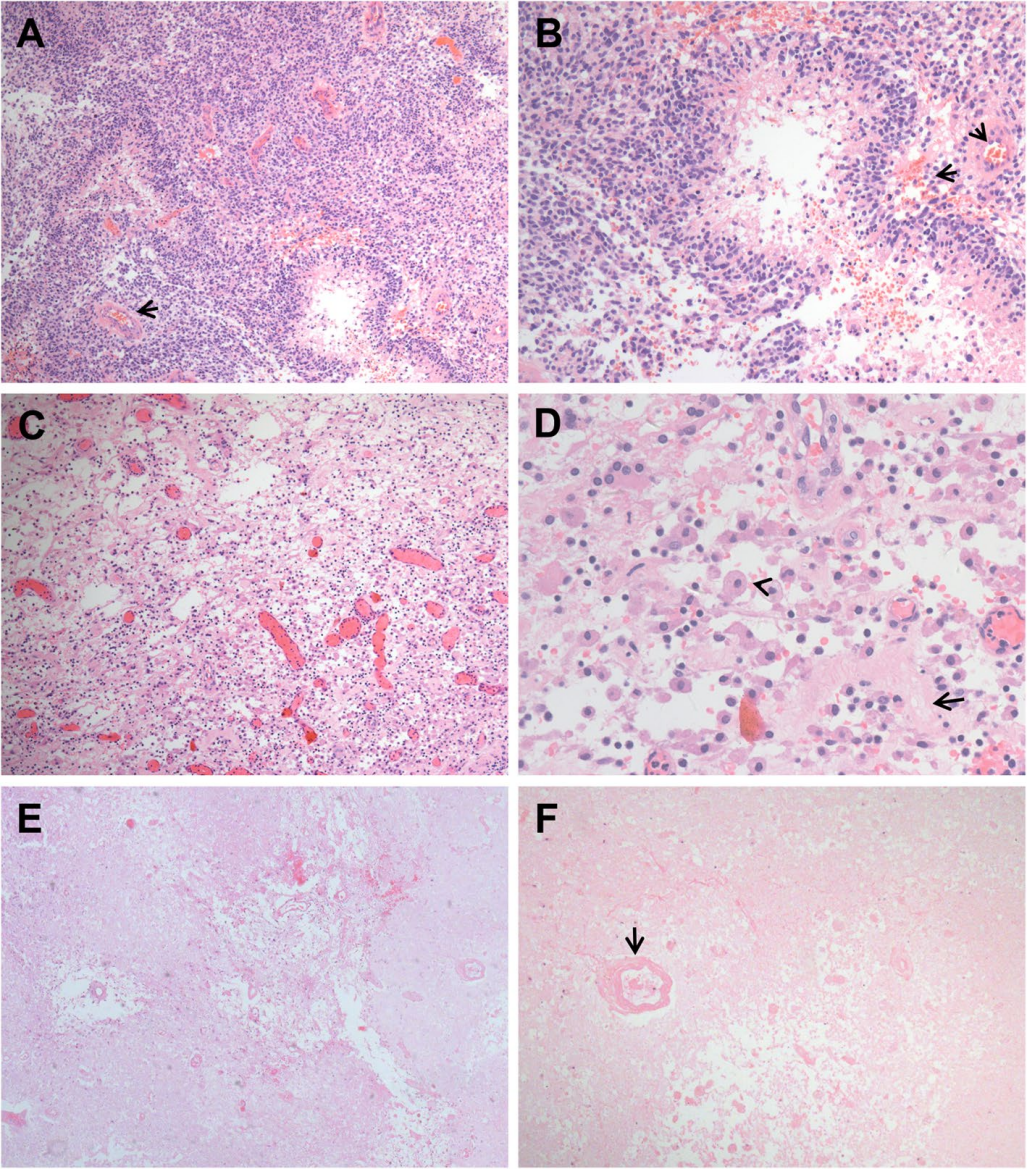
Histopathology types of recurrent glioma. **A**,** B**, Activie morphology of recurrent gliomas characterized by healthy-looking tumor cells exhibiting solid growth, more dense cellularity, and often with microvascular proliferation (arrows), indicating robust tumor recurrence and even increased tumor grade. **C**,** D**, Low activity tumor cells in low density and the tumor cells still may be alive, but do not appear healthy (arrowhead). Such cells usually show very few mitoses, if any. Blood vessels often appear devitalized, hyalinized, and distorted (arrow). These are considered as evidence of after treatment changes, not expressions of recurrent or residual active high grade tumor. **E**,** F**, No activity or radiation necrosis means the tumor cells were not seen, mainly necrotic tissue including coagulation necrosis and fibrinoid necrosis of vessels (arrow), which are typical feature of treatment effect. (Magnifications, A, 100 × ; B, 200 × ; C, 100 × ; D, 400 × ; E, 40 × ; F, 100 ×)

Pathology diagnoses of primary glioma and recurrent glioma were reviewed by two pathologists (XS.Y. and J.Z.) who were blinded to the clinical outcomes. Their consistency can reach 95.6%, and the inconsistent ones will be discussed until reached consensus conclusion. The histopathological diagnosis of the first operation was made according to the 2016 WHO CNS criterion [6], and the histopathological features of secondary operations were classified into three types (Fig. 1), mainly referring to the description of Haider and Woodworth about the activity of tumor cells [7, 8], as follows (1): active group, active tumor cells without degeneration or showing more atypical architecture, such as irregular nuclei or more mitosis; (2) low-activity group, tumor cells present with treatment-related changes, such as degeneration, coagulation necrosis and apoptosis, and vascular changes, such as fibrinoid necrosis; (3) no activity/necrosis group, typical tumor cells absent and hyalinized vessels and necrosis present. In addition, molecular pathological status, including the Ki67 index and isocitrate dehydrogenase 1 (IDH1) mutation status, was recorded via a review of molecular pathology records. Mutational status of IDH1 was determined using the Sanger technique.

### Statistical methods

All statistical analyses were performed using SPSS Statistics version 24.0 (IBM, Armonk, New York) or GraphPad Prism version 8.0.1 (GraphPad Software, San Diego, California). Continuous variables are described as the mean ± standard deviation (SD) or median with range. Differences between three histopatologic groups of reoperation were compared using One-Way ANOVA for continuous variables and Fisher's exact test for categorical variables with a small sample size. The Kaplan–Meier method was used to estimate overall survival for each group, and a log-rank test was performed to determine statistically significant differences between groups. To assess the relative impact of multiple variables on overall survival after reoperation, a multivariate Cox proportional hazards model was used. Given the relatively small cohort size, and some missing data concerning chemotherapy or radiotherapy information, only the IDH1 status, patient age, WHO grade of the first operation and histopatologic groups of reoperation are included in the model because most of they are well-known prognostic factors of glioma patients. The threshold for statistical significance was set at *P* < 0.05.

## Results

### Patients

In this study, 89 glioma patients were reviewed, including 52 males and 37 females. The histopathological results of the first operation were 31 cases of WHO grade 2 (including 28 cases of astrocytomas and 3 of oligodendrocyte glioma), 17 cases of WHO grade 3 (including 14 cases of anaplastic astrocytoma and 3 of anaplastic oligodendrocyte glioma), and 41 cases of WHO grade 4 (all glioblastoma). The average age of the patients at the first operation was 41.34 ± 13.71 years. Most patients (83, 93.33%) underwent a single reoperation for recurrent disease; 6 (6.74%) patients received a third surgery (Table 1). All patients included had supratentorial gliomas, and compared with the first imaging data, all patients were considered to have in situ tumor recurrence. The extent of tumor resection at the primary surgery achieved GTR in 72 cases and PR in 17 cases.

**Table 1:** Cohort characteristics at first operation

Total	89	100%
Age < 50 yr	58	65.2%
≥ 50 yr	31	34.8%
Gender Male	52	58.4%
Female	37	41.6%
WHO Grade 2	31	34.8%
3	17	19.1%
4	41	46.1%
IDH1 WT	36	40.4%
Mu	19	21.3%
NA	34	38.2%
Radiotherapy Yes	68	76.4%
No	15	16.9%
NA	6	6.7%
Chemotherapy Yes	39	43.8%
No	15	16.9%
NA	35	39.3%
Second reoperation (%)	6	6.7%
KPS < 80	9	10.1%
≥ 80	80	89.9%
Extent of resection GTR	72	80.9%
PR	17	19.1%

Abbreviations: *WHO* world health organization, *WT* wild type, *Mu* mutant, *NA* not available, *KPS* Karnofsky performance status, *GTR* gross total resection, *PR* partial resection

After the first operation, 68 patients received radiotherapy, and 39 patients received chemotherapy (Table 1), including 6 who received radiotherapy alone, 36 who received radiotherapy combined with chemotherapy and 3 who received chemotherapy alone. Nine patients did not receive any adjuvant treatment following the primary surgery, while adjuvant treatment information for radiotherapy and chemotherapy was not available in 6 and 35 cases, respectively. The median follow-up time from primary resection was 29.17 months (range, 6.77—194.87 months), and the median follow-up time after reoperation was 7.07 months (range, 1.33—173.90 months). The median interval time between two operations was 13.97 months (range, 3.02—178.53 months). Following the second operation, 10 patients received radiotherapy or concurrent chemo-radiation treatment, whereas 15 patients only received chemotherapy and/or targeted therapy.

### WHO grade of primary glioma and clinical outcomes

**Fig. 2:**
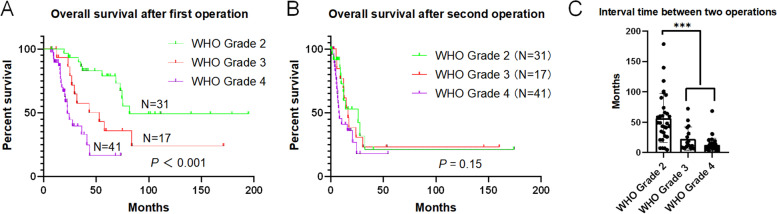
WHO grade of the first operation pathology was related to the patients’ survival time after the first operation (**A**), but there was no significant statistical differences with the survival time after the re-operation (**B**). The interval time between the two operations of primary high-grade glioma patients was shorter than low grade glioma (WHO grade 2) ( ***, *P* < 0.001) (**C**)

By univariate analysis, we found that the tumor primary pathology grade correlated with survival time following the first operation but that there was no significant association of the primary tumor grade with survival time after reoperation (Fig. 2A and B). The interval time between the primary and recurrent surgery was shorter in primary high-grade glioma patients than in low-grade glioma (WHO grade 2) patients (*P* < 0.001) (Fig. 2C). Among the 31 primary WHO grade 2 glioma patients, 22 (71.0%) had an increased tumor grade to high-grade glioma when they experienced relapse; the interval between the two operations was longer than that in 9 patients (29.0%) whose tumor grade did not increase, though there was no significant difference (64.74 ± 8.85 months vs. 38.04 ± 10.51 months, *P* = 0.09).

### IDH1 status and clinical prognosis

**Fig. 3:**
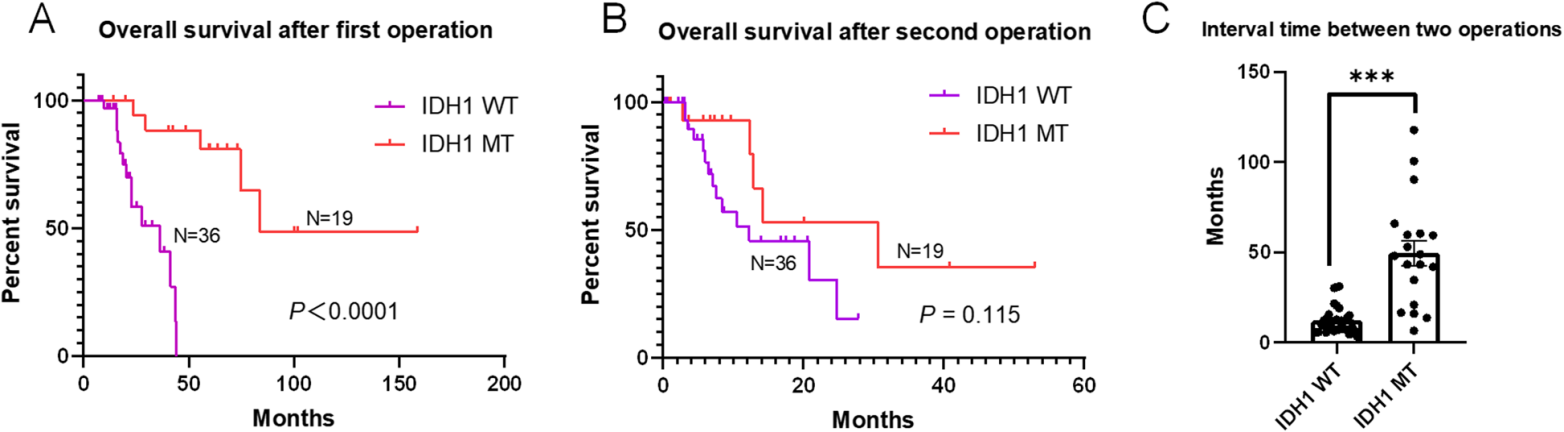
IDH1 mutant (MT) glioma patients were significantly correlated with the longer survival time after the first operation than IDH1 wild-type (WT) patients (**A**). However, there was no significant difference between the status of IDH1 and the patients’ survival time after the second operation (**B**). The interval time between two operations in IDH1 MT patients was also significantly longer than that in IDH1 WT patients( ***, *P* < 0.001) (**C**)

Fifty-five patients had IDH1 status records, with 36 having wild-type tumors and 19 IDH1 R132 mutations. Of the 36 IDH1 wild-type tumor patients, the pathology results of the primary surgery diagnosed 31 cases (86.11%) as GBM, 2 cases (5.56%) as WHO grade 2, and 3 cases (8.33%) as WHO grade 3. Of the 19 IDH1 mutant tumor patients, 14 cases (73.68%) were WHO grade 2, 4 cases (21.05%) were WHO grade 3, and 1 case (5.26%) was grade 4. Univariate analysis showed that compared to IDH1 wild-type glioma, IDH1-mutant glioma correlated significantly with a longer survival time after the first operation. However, there was no significant difference between the status of IDH1 and survival time after the second operation (Fig. 3A and B). The interval time between the two operations was also significantly longer in IDH1 mutant tumor patients than in IDH1 wild-type tumor patients (Fig. 3C).

### Histopathological patterns of recurrent glioma and clinical outcomes

Three histopathological types of recurrent gliomas from 89 cases were analyzed, classifying 67 cases (75.28%) into the active group, 14 (15.73%) into the low-activity group, and 8 (8.99%) into the necrosis group. There were significant differences in the radiation dose, chemotherapy status and interval time between the two operations in these three groups. Different from the increase of Ki67 index change in the active group, Ki67 index change decreased in both necrosis group and low-activity group (Table 2).

**Table 2:** Clinical characteristics according to histopathologic parameters of recurrent glioma

N	Active group	Low activity group	Necrosis group	*P*
	67	14	8	
Age (mean ± SD)	41.27 ± 13.58	41.86 ± 15.52	41.00 ± 7.35	0.987
Gender Male	38	6	2	0.169
Female	29	8	6	
WHO Grade 2	26	3	2	0.366
3	10	4	3	
4	31	7	3	
IDH1 R132 WT	27	5	4	0.460
Mu	17	1	1	
NA	23	8	3	
Radiotherapy Yes	48	12	8	0.465
No	14	1	0	
NA	5	1	0	
Radiation dose (Gy)	58.44 ± 0.61	60.46 ± 1.16	63.75 ± 3.75	0.031^*^
Chemotherapy Yes	25	7	7	0.044^*^
No	11	4	0	
NA	31	3	1	
interval time (Mon.)	34.93 ± 4.37	17.30 ± 4.09	12.54 ± 3.76	0.051
Ki67 change	11.08 ± 4.50	-6.5 ± 11.50	-12.75 ± 5.12	0.184

One-Way ANOVA test for continuous variables and Fisher's exact test for categorical variable

Abbreviations: *WHO* world health organization, *WT* wild type, *Mu* mutant, *NA* not available, *Mon* month

**Fig. 4:**
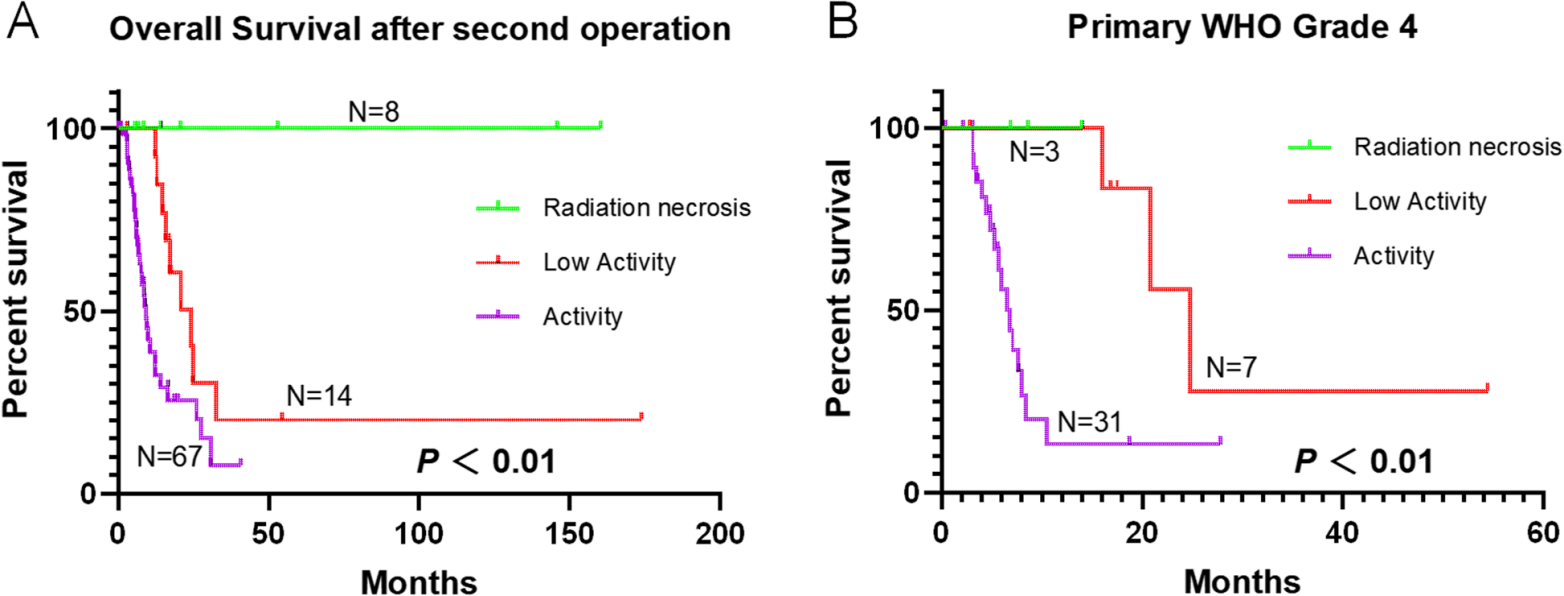
Histopathological patterns of recurrent gliomas were related to the overall survival of glioma patients after re-operation. Necrosis indicated a better prognosis (**A**), as did necrosis and low activity in primary GBM patients (**B**)

Eight patients in the necrosis group were still alive at the last follow-up date, with an average follow-up time of 51.84 months after reoperation. The median survival time after reoperation was 24.13 months in the low-activity group and 9.50 months in the active group. Due to the small sample size of necrosis group and low activity group, we combined these two groups into one group into multivariate Cox analysis for comparison with active group. Similarly, we combined primary WHO grade 3 and 4 into high grade group and grade 2 as low grade group into multivariate Cox model. Finally, we found that the histopathological patterns of recurrent gliomas were related to survival time after reoperation (Fig. 4 and Table 3), but the patient age, primary WHO grade, and IDH1 status were not significantly related to the survival time after reoperation.

**Table 3:** Cox regression analysis of the survival of glioma patients after re-operation

	HR	95.0% HR CI		*P*
		Lower	Upper	
IDH1 (WT / Mu)	1.550	0.328	7.320	0.580
Age (< 50 / ≥ 50)	1.450	0.518	4.064	0.479
Primary WHO Grade (Low Grade / High Grade)	0.287	0.043	1.929	0.199
Histopathologic parameters of recurrent glioma (Active / Low activity or Necrosis)	6.810	1.730	26.814	0.006^*^

Abbreviations: *WHO* world health organization, *Mu* mutant, *WT* wild type

## Discussion

Recurrence of glioma is often inevitable, and reoperation is still an important method to treat recurrent glioma. Glioma patients can obtain survival benefits from surgery even though resection is often incomplete due to the invasive nature of glioma [9, 10]. Some patients may even undergo a third or more surgical treatments. In the present cohort, the proportion of patients who received three operations was 6.5%, which was lower than the 16.7% reported by Ringel, who only included patients with GBM [11]. The pathology of recurrent glioma showed obvious heterogeneity; not only did the molecular characteristics change of the tumor, but the histopathology also changed due to previous treatment as well as tumor progression. Some studies have found that the molecular state of glioma changes upon recurrence. Hulsebos found that low-grade glioma patients with increased pathological grade after recurrence harbored more gene mutations than patients with an unchanged pathological grade [5]. Mukasa confirmed a large number of new mutations in recurrent gliomas, with only a limited number of key driving genes remaining unchanged [12]. Nandeesh found that recurrent GBM expressed more EGFR and tumor stem cell-related genes but that p53 and IGFBP-3 expression was unaltered in paired samples [13]. Therefore, relapsed glioma shows significant heterogeneity in time and acquires no stereotypical mutations, obvious "time heterogeneity" [14].

However, unlike the rapid development of molecular pathology, the histopathological diagnosis of recurrent glioma is still controversial, especially for patients after radiation therapy(RT) and temozolomide (TMZ) chemotherapy. Some patients with low-grade gliomas develop high-grade gliomas, while the activity of tumor cells may decrease in some high-grade gliomas when second operation. The components of recurrent glioma may contain tumor cells with different activity levels and nonneoplastic brain elements with reactive changes. Therefore, conventional pathology classification may not be suitable for the diagnosis of recurrent glioma. According to our experience and reports from the literature, neuropathologists sometimes do not report the WHO grade of recurrent glioma, especially for glioma received adjuvant treatment, and they prefer to report descriptive diagnoses, such as “changes after treatment”, “recurrent/residual high-grade glioma with treatment effect” and “treatment-related effects” [7]. In a few cases, radiation necrosis has also been diagnosed in the absence of obvious tumor cells and with only necrotic tissue. Some articles have also reported that treatment-related changes in recurrent gliomas correlate with the prognosis of patients [8, 15]. For further analysis, we classified recurrent gliomas into three types according to histopathological findings of tumor cell activity and necrosis degree in reoperation samples. In this cohort study, most of the reoperation samples contained active and healthy tumor cells, though a small number of cases showed unhealthy and low-activity tumor cells. Nevertheless, only necrotic tissues without obvious tumor cells were found in some cases, which was diagnosed as radiation necrosis. The histopathological type of recurrent glioma is related to previous radiotherapy, with a tendency toward tissue necrosis and a "decline" in tumor cell activity/tumor grade. Patients without radiotherapy may be more likely to have active tumor cells, a higher Ki67 index, and an increased pathological tumor grade. Therefore, for patients with recurrent glioma indicated by imaging, the previous treatment is also an important consideration when evaluating reoperation, and there is still a certain proportion of patients with reoperation were radiation necrosis tissue rather than real tumor recurrence.

Our further analysis showed that the WHO grade or IDH1 status of tumors from the primary surgery is related to patient OS following the first operation, while there is no significant correlation with survival time after reoperation. The time interval between the two operations was significantly longer in patients with low-grade and/or IDH1 mutation glioma than that with high-grade and/or IDH1 wild-type glioma, indicating that the survival benefit of low-grade glioma and/or IDH1 mutation mainly appeared on late recurrence. Once glioma relapses, primary low grade or IDH1 mutation may not nesissary prognostic factors, as revealed in our study that there is no significant difference in survival time as compared with primary high grade and IDH1 wild-type glioma after reoperation. However, we found the histopathological characteristics of recurrent tumors to be related to the survival of patients with recurrence following reoperation, and multivariate Cox regression analysis showed that it is an independent prognostic factor. This result is similar to some other studies showing that the presence of histologically confirmed viable active tumor cells is significantly associated with unfavorable prognosis [8, 16]. Considering the decrease of Ki67 index change in necrosis group and low-activity group, our results are also similar to Gzell et al. reported that change in Ki67 can predict survival in patients having repeat craniotomy within 6 months of radiotherapy for high-grade glioma [17]. Reduced activity of residual tumour cells and widespread necrosis is important for better prognosis of patients.

As the histopathology of recurrent glioma can indicate the effect of previous treatment and the patient's prognosis after reoperation, we believe that the three types of histopathological classification can guide subsequent treatment of recurrent glioma. If the histopathology indicates necrotic changes after treatment and no tumor cell is found, we need to consider whether the surgical tissue is representative. If there is no other evidence indicating the existence of tumor recurrence, the previous treatment was likely effective, and most of these patients have a good prognosis. Thus, we can consider no further antitumor treatment but close follow-up. For example, 8 cases in the "necrosis group" in our study all had a history of high-dose radiotherapy, accompanied by a short interval between the two operations. We consider that our previous treatments, especially radiotherapy, were effective, and only close follow-up after reoperation was applied. For the low-activity group, this result suggests that previous treatment may at least have reduced the activity of tumor cells. For such cases, ensuing treatment should be considered "relatively mild", despite the presence of low-activity tumor cells is found on histopathology. We usually recommend chemotherapy, which can be the previous scheme or a different protocol. The activity group shows the most common type of recurrence pattern, and most of these patients had an increased pathological grade and Ki67 index. Some of the patients did not receive adjuvant treatment (radiotherapy and chemotherapy). Therefore, it is necessary to implement active antitumor strategies, referring to previous treatments, including concurrent chemoradiotherapy, reradiotherapy, and/or chemotherapy with altering regimens.

## Limitations

First, criteria for reoperation for patients with recurrent glioma may varied among doctors and medical centers. In addition, as the number of cases in this study was small, a larger sample multicenter retrospective study is needed for verification. Second, the relative proportions of active tumor and treatment effects varied considerably, being low in some and high in others. As these were not easy to quantify, this study did not refine their proportion or degree. Furthermore, the presence of only low-grade astrocytoma was not easy to distinguish from low-activity tumors; thus, such patients may have been included in the low-activity group. Third, the pathological diagnosis of gliomas has undergone great changes and modern molecular characteristics of tumors has become an important and difficult aspect of clinical practice [18]. Due to the incomplete molecular pathology except IDH1 in this cohort study, no further analysis in that regard was performed. Other factors that may be related to prognosis, such as tumor volume and chemotherapy duration, are not included in this study. We focused on analyzing the clinical significance of recurrent glioma histopathology.

## Conclusion

The histopathology of recurrent glioma is related to previous treatment and the interval time between the two operations. Radiotherapy and a higher radiation dose may be associated with necrosis or low activity of recurrent glioma. Analysis of the histopathology of recurrent glioma is helpful for predicting the prognosis of recurrent patients and suggesting subsequent treatment strategies.

## Data Availability

The datasets used and/or analysed during the current study available from the corresponding author on reasonable request.
